# Pharmacodynamic properties for inhibition of cAMP- and cGMP elimination by pentoxifylline remain unaltered in vitro during hypothermia

**DOI:** 10.1186/s13049-022-01060-y

**Published:** 2022-12-15

**Authors:** Anders Lund Selli, Adrina Kalasho Kuzmiszyn, Natalia Smaglyukova, Timofey Kondratiev, Ole-Martin Fuskevåg, Georg Sager, Erik Sveberg Dietrichs

**Affiliations:** 1 Department of Medical Biology, Experimental and Clinical Pharmacology, UiT – The Arctic University of Norway, Tromsø, Norway; 2 Research and Development Department, Norwegian Air Ambulance Foundation, Oslo, Norway; 3 Center for Psychopharmacology, Diakonhjemmet Hospital, Oslo, Norway; 4 Anesthesia and Critical Care Research Group, Department of Clinical Medicine, UiT - The Arctic University of Norway, Tromsø, Norway; 5 Division of Diagnostic Services, Department of Laboratory Medicine, University Hospital of North Norway, Tromsø, Norway; 6 Division of Surgical Medicine and Intensive Care, University Hospital of North Norway, Tromsø, Norway

**Keywords:** Hypothermia, PDE-inhibitors, Cyclic AMP, Cyclic GMP, Blood viscosity, Inotropy, Afterload reduction, Cardiovascular dysfunction

## Abstract

**Background:**

Rewarming from hypothermia is associated with severe complications, one of which is hypothermia-induced cardiac dysfunction. This condition is characterized by decreased cardiac output accompanied by increased total peripheral resistance. This contributes to mortality rate approaching 40%. Despite this, no pharmacological interventions are recommended for these patients below 30 °C. Raising the intracellular levels of cAMP and/or cGMP, through PDE3- and PDE5-inhibitors respectively, have showed the ability to alleviate hypothermia-induced cardiac dysfunction in vivo. Drugs that raise levels of both cAMP and cGMP could therefore prove beneficial in patients suffering from hypothermia-induced cardiac dysfunction.

**Methods:**

The unselective PDE-inhibitor pentoxifylline was investigated to determine its ability to reach the intracellular space, inhibit PDE3 and PDE5 and inhibit cellular efflux of cAMP and cGMP at temperatures 37, 34, 30, 28, 24 and 20 °C. Recombinant human PDE-enzymes and human erythrocytes were used in the experiments. IC_50_-values were calculated at all temperatures to determine temperature-dependent changes.

**Results:**

At 20 °C, the IC_50_-value for PDE5-mediated enzymatic breakdown of cGMP was significantly increased compared to normothermia (IC_50_: 39.4 µM ± 10.9 µM vs. 7.70 µM ± 0.265 µM, *p*-value = 0.011). No other significant changes in IC_50_-values were observed during hypothermia.

**Conclusions:**

This study shows that pentoxifylline has minimal temperature-dependent pharmacodynamic changes, and that it can inhibit elimination of both cAMP and cGMP at low temperatures. This can potentially be effective treatment of hypothermia-induced cardiac dysfunction.

*Trial registration*: Not applicable.

## Background

Accidental hypothermia is defined as an involuntary drop in body core temperature to < 35 °C and is associated with multiple complications, such as hypothermia-induced cardiac dysfunction (HCD) [1]. This contributes to the high mortality of accidental hypothermia estimated to be between 20 and 40% [2, 3]. In addition to being an accidental condition, hypothermia is also used therapeutically. Patients suffering from cardiac arrest are cooled to 32–36 °C after successful resuscitation, during targeted temperature management (TTM) [4], thought to be beneficial mainly due to avoidance of hyperpyrexia but each °C of temperature reduction below 35 °C does also reduce brain metabolism by ~ 7% [5]. Moreover, HCD is a threat in patients treated with TTM [6] and pharmacological interventions should be explored to increase survival in these two patient groups.

The underlying mechanisms for HCD have been largely unknown. Recent animal studies have suggested that cardiac output (CO) reduction is related to increased total peripheral resistance (TPR) [7, 8]. In vivo experiments testing epinephrine, traditionally used to elevate CO in emergency and critical care medicine, have shown reduced inotropic effect when used during hypothermia [9, 10]. However, the phosphodiesterase 3 (PDE3)-inhibitors milrinone and levosimendan had maintained ability to increase CO and reduce TPR during hypothermia, thus preventing HCD [11, 12].

PDE3-inhibitors impede the enzymatic breakdown of cAMP, causing raised intracellular levels of this cyclic nucleotide and activation of PKA. As the PDE3-enzyme is found both in the heart and in vascular smooth muscle, inhibitors cause increased cardiac inotropy and peripheral vasodilation, which in turn decreases TPR [13]. In a previous experiment, we showed that PDE3-inhibitors milrinone, amrinone and levosimendan inhibit the PDE3-enzyme in therapeutic concentrations down to 20 °C [14].

Reduction of TPR could also be achieved through increased smooth muscle cell-levels of cGMP, which is metabolized by phosphodiesterase 5 (PDE5). cGMP-elevation has also proved a promising strategy during hypothermia, indirectly increasing CO, by reduction of TPR [8]. PDE5-inhibitors sildenafil and vardenafil have shown ability to inhibit the PDE5-enzyme and increase cGMP in therapeutic concentrations down to 20 °C [15].

In addition to enzymatic elimination of cAMP and cGMP, mainly through PDE3 and PDE5, cAMP and cGMP are also eliminated by efflux pumps in the cell membranes [16, 17]. The efflux of cAMP is primarily through ATP-binding cassette subfamily-C 4 (ABCC4) and cGMP through ABCC5 [18]. We therefore hypothesized that an unselective inhibitor of both PDE3, PDE5, ABCC4 and ABCC5 could be beneficial in treatment of HCD. Accordingly, we investigated the ability of pentoxifylline, an unselective PDE-inhibitor that is in clinical use, to reach its intracellular site of action and inhibit PDE3 and PDE5, as well as ABCC4 and ABCC5, during hypothermia.

## Materials and methods

Pentoxifylline (Sigma-Aldrich, St. Louis, MO, USA) was used in all experiments. Detailed methods for assessing intracellular access, enzyme inhibition and cellular efflux were first described in earlier publications [14, 15].

### Temperatures

According to the European Resuscitation Council, hypothermia is divided into mild (35–32 °C), moderate (32–28 °C) and severe (below 28 °C) [19]. To get a broad view of the pharmacodynamics of pentoxifylline, we included several temperatures ranging from normothermia through all stages of hypothermia. The included temperatures were 37 °C, 34 °C, 32 °C, 28 °C, 24 °C and 20 °C.

### Intracellular access

A review of the literature was performed to get an estimate of relevant therapeutic concentration of pentoxifylline. We searched PubMed with the following mesh terms: (Pentoxifylline) AND (intravenous) AND (plasma concentration) OR (serum concentration). The articles had to report adult human data and cardiovascular condition to be considered. Pentoxifylline was incubated at a final concentration of 100 µM as this corresponded to the highest concentrations found in the review [14, 15].

Blood was provided by Blodbanken UNN (Department of Immunohematology and Transfusion Medicine, University Hospital of North Norway) where all participants (n = 18) were pre-screened and only admitted as donors if they were healthy. Each parallel only included blood from one donor. Experiments were initiated by washing and centrifuging recently (< 24 h) drawn EDTA-blood 3 times with Krebs–Ringer-Phosphate-Buffer containing glucose (KRPB/G, pH ~ 7,4). Final Hct was of 0.40 in the incubate solution. Blood suspension was added to tubes containing 50 µL of either pentoxifylline or MQ-water (negative control). Each experiment contained triplicates of both drugs and control, and 3 experiments at each temperature were conducted – in total 9 parallels. The reactions were stopped after 30 min by putting the tubes on ice and adding 4 mL ice cold KRPB/G. The solutions were washed and centrifugated 3 times with ice cold KRPB/G. 50 µL of the remaining solution was then added to Eppendorf tubes along with 50 µL internal standard (IS), containing [13C,2H3]-Pentoxifylline, 50 nM (Alsachim, Illkirch Graffenstaden, France). 5 samples contained 50 µL known concentrations of pentoxifylline, and 50 µL IS, and served as controls for accurate analysis. All samples were added 200 mL 0.1 M ZnSO_4_, to lyse the erythrocytes, and then centrifugated. 30 µL was taken from Eppendorf tubes for measurements of protein concentration before adding 500 µL acetonitrile. 100 µL from each tube was collected for analysis using mass spectrometry (MS) [14, 15].

### Enzyme inhibition

Assessment of pentoxifyllines ability to inhibit the phosphodiesterase enzymes were performed by incubating cAMP or cGMP with seven different concentrations of pentoxifylline. The concentrations were increasing by a factor of 10 ranging from 1.00 nM to 1.00 mM. For PDE3 assessment, the incubation solution included cAMP and for PDE5 assessment it included cGMP. Experiments were performed in triplicates each day at three separate days - in total 9 parallels. The reaction was started by adding either a solution containing 0.016 units/µg protein of PDE3 (Abcam, Cambridge UK), or 0.022 units/µg protein of PDE5 (Sigma-Aldrich, St. Louis, USA), to the Eppendorf tubes. Control samples were free of drug and was either with or without PDE3 or PDE5. This was done to assure that only the relevant PDE was responsible for breakdown of the cyclic nucleotide, as no other enzyme nor cellular material was added to the incubations. The incubation time was 30 min. Reaction was stopped by adding methanol to the tubes. Internal standard of cGMP/GMP or cAMP/AMP (Sigma-Aldrich, St. Louis, MO, USA, Germany and Toronto Research Chemicals Inc., Ontario, Canada) were added to each sample. 5 samples contained only known concentrations of cGMP/GMP or cAMP/AMP and served as calibrators. Samples were analyzed for cGMP/GMP and cAMP/AMP content, using MS [14, 15].

### Cellular efflux inhibition

Cellular efflux was estimated with inside-out vesicles (IOV)s where erythrocytes from healthy, human donors were sampled. Donors were pre-screened and only admitted as donors by Blodbanken UNN (Department of Immunohematology and Transfusion Medicine, University Hospital of North Norway) if they were healthy. The erythrocytes were separated from plasma by centrifugation and washed. Inside-out vesicles were prepared according to Orvoll et al. [20] with minor modifications. Percentage IOV was verified using acetylcholinesterase accessibility test [21]. Batches of IOVs used in the parallels were made 8 times, including blood from a total of 35 healthy donors [14, 15].

IOVs were then incubated with or without 2 mM ATP and 7 different concentrations of pentoxifylline increasing by a factor of 10 ranging from 1.00 nM to 1.00 mM. The incubation solutions also included radioactive labeled [^3^H]-cGMP or [^3^H]-cAMP (Perkin Elmer, Boston, MA, USA), at a concentration of respectively 2 µM and 20 µM. [^3^H]-cAMP was used to assess ABCC4-inhibition and [^3^H]-cGMP for ABCC5-inhibition. The assays were performed in triplicates at 3 different days: In total 9 parallels were performed to calculate results for each concentration of pentoxifylline at all temperatures. Incubation time of 60 min was chosen to ensure sufficient quality of the samples for each parallel. The transport was stopped by adding ice cold buffer. The IOVs were then filtered through a nitrocellulose membrane (Bio-Rad Laboratories, Feldkirchen Germany), and the membrane was dried. The dried membranes were later added scintillation fluid and radioactivity was measured using a Packard TopCount NXT (Packard, Downers Grove, IL, USA) [14, 15].

### Mass spectrometry (MS) analysis

Quantification of cAMP/AMP, cGMP/GMP and pentoxifylline in PDE- and intracellular access experiments were performed with liquid chromatography tandem mass spectrometry (LC–MS/MS). Preparation of samples for LC–MS/MS-analysis is described in paragraphs above. The method was found to be linear from 0.2 nM to at least 2000 nM (r^2^ > 0.998) for cAMP, cGMP and AMP. For GMP the method was linear from 2 nM to at least 2000 nM (r^2^ > 0.998), and 10 nM to at least 5000 nM for pentoxifylline (r^2^ > 0.998). Lower limit of quantification (LLOQ) was found to be 0.2 nM for cAMP, cGMP and AMP, 2 nM for GMP and 10 nM for pentoxifylline (2 µl injection volume) [14, 15].

### Data analysis

Analysis and graph production were performed in SigmaPlot 14.0 (Systat Software, San Jose, CA, USA.). Ability to inhibit the different elimination pathways, enzymes and efflux pumps, were calculated as IC_50_-values, as according to Chou [22]. Ki-values were obtained by using the methods described by Cheng and Prustoff [23]. Measurement of intracellular concentrations of drugs were adjusted for protein concentrations in each sample. The incubation concentrations were also adjusted for protein concentration in each sample to evaluate the access in percentage. Analysis of variance (ANOVA) with Holm-Sidak multiple comparison post-hoc test was performed to evaluate changes in IC_50_-values compared to baseline (37 °C) for all elimination pathways. The same analysis was performed for intracellular concentrations of pentoxifylline during hypothermia compared to baseline (37 °C). When data was not normally distributed ANOVA on ranks and Dunn`s post hoc test was performed. Data are presented as mean ± standard error of the mean (SEM). *p*-values were considered significant when < 0.05.

## Results

### Intracellular access

Pentoxifylline incubated at a final concentration of 100 µM was able to reach its intracellular site of action at all temperatures down to 20 °C. No significant changes were detected between temperatures for either absolute intracellular drug concentration in nmol/g or percent (%) of added pentoxifylline concentration per gram protein. (Table 1).

**Table 1:** Values depicting intracellular availability of pentoxifylline at temperatures ranging from 37 to 20 °C

Intracellular availability	Pentoxifylline (nmol/g protein)	Pentoxifylline [% of added (drug)/g protein]
37 °C	17.7 ± 3.07	0.15 ± 0.021
34 °C	11.5 ± 0.755	0.15 ± 0.005
32 °C	21.5 ± 3.28	0.15 ± 0.018
28 °C	15.2 ± 2.19	0.15 ± 0.021
24 °C	31.5 ± 8.22	0.13 ± 0.018
20 °C	12.1 ± 3.07	0.12 ± 0.011

Values are mean ± SEM. No significant change from 37 °C was detected for either absolute concentration nor %-difference

### Phosphodiesterase-activity

**Fig. 1:**
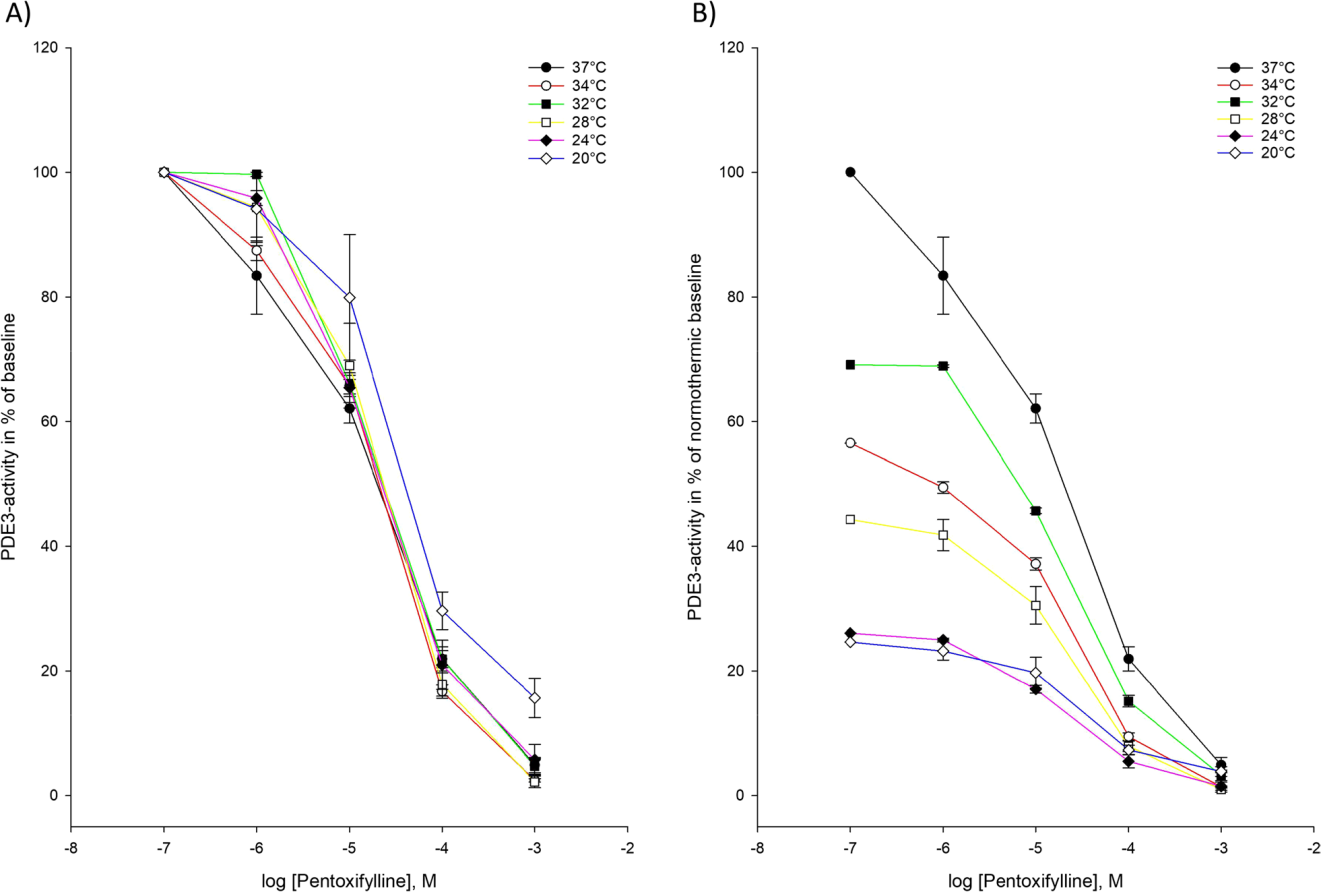
Temperature-dependent inhibition of phosphodiesterase-3 (PDE3) by pentoxifylline **A** Pentoxifylline inhibition curves for PDE3-activity at temperatures ranging from 37 to 20 °C. The doses of pentoxifylline are in logarithm of the concentration in mol/L. **B** Inhibition curves for PDE3-activity by pentoxifylline in % of normothermic inhibition curve at temperatures ranging from 37 to 20 °C. The doses of pentoxifyllne are in logarithm of the concentration in mol/L

**Fig. 2:**
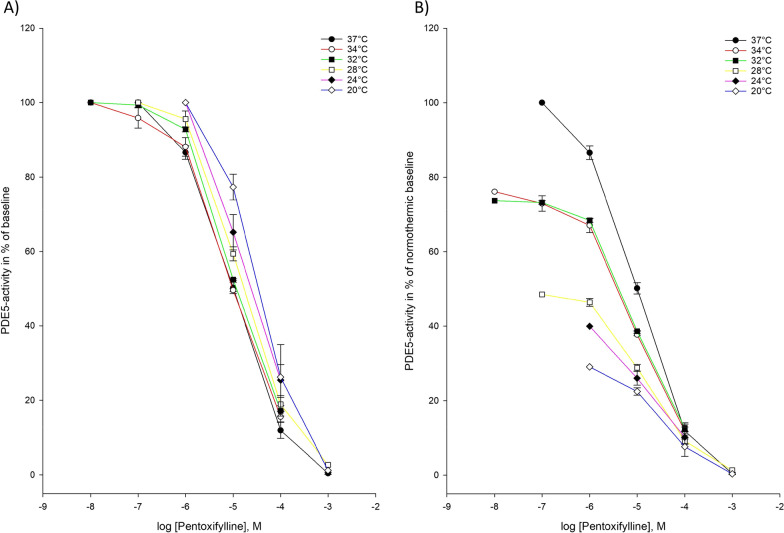
Temperature-dependent inhibition of phosphodiesterase-5 (PDE5) by pentoxifylline **A** Pentoxifylline inhibition curves for PDE5-activity at temperatures ranging from 37 to 20 °C. The doses of pentoxifylline are in logarithm of the concentration in mol/L. **B** Inhibition curves for PDE5-activity by pentoxifylline in % of normothermic inhibition curve at temperatures ranging from 37to 20 °C. The doses of pentoxifyllne are in logarithm of the concentration in mol/L

PDE3-activity was inhibited at all temperatures ranging from 37 to 20 °C. No statistically significant changes were observed for IC_50_ – and Ki-values during hypothermia (Tables 2, 3) (Figs. 1, 2).

**Table 2:** IC_50_-values for inhibition of phosphodiesterase-3 (PDE3), phosphodiesterase-5 (PDE5), and inhibition of cAMP- and cGMP-efflux at temperatures ranging from 37 to 20 °C

Temperature	PDE3	PDE5	cAMP-efflux	cGMP-efflux
37 °C	15.8 ± 4.25	7.70 ± 0.265	1.33 ± 0.566	0.531 ± 0.338
34 °C	14.2 ± 1.51	10.7 ± 1.92	3.61 ± 2.59	0.893 ± 0.401
32 °C	26.7 ± 4.70	15.5 ± 0.753	2.01 ± 0.819	0.724 ± 0.206
28 °C	22.4 ± 6.36	24.3 ± 5.58	1.93 ± 1.11	0.851 ± 0.281
24 °C	29.2 ± 4.72	25.2 ± 6.57	–	0.502 ± 0.315
20 °C	31.0 ± 9.47	39.4 ± 10.9*	–	0.388 ± 0.070

Values are mean ± SEM and given in µM. * Significant difference (*p*-value < 0.05), when compared to normothermic baseline. No inhibition of ABCC4-activity was detectable below 28 °C

**Table 3:** Ki-values for inhibition of phosphodiesterase-3 (PDE3), phosphodiesterase-5 (PDE5), and inhibition of cAMP- and cGMP-efflux at temperatures ranging from 37 to 20 °C

Temperature	PDE3	PDE5	cAMP-efflux	cGMP-efflux
37 °C	0.723 ± 0.195	1.95 ± 0.067	0.805 ± 0.343	0.300 ± 0.191
34 °C	0.651 ± 0.069	2.71 ± 0.487	2.03 ± 1.46	0.505 ± 0.226
32 °C	1.22 ± 0.215	3.94 ± 0.191	1.16 ± 0.526	0.409 ± 0.116
28 °C	1.02 ± 0.291	6.17 ± 1.42	0.918 ± 0.465	0.481 ± 0.159
24 °C	1.34 ± 0.216	6.40 ± 1.67	–	0.320 ± 0.183
20 °C	1.42 ± 0.434	10.0 ± 2.78*	–	0.602 ± 0.174

Values are mean ± SEM and given in µM. * Significant difference (*p*-value < 0.05), when compared to normothermic baseline. No inhibition of ABCC4-activity was detectable below 28 °C

During hypothermia down to 20 °C pentoxifylline was able to inhibit PDE5-mediated breakdown of cGMP. At 20 °C, the IC_50_-values, and Ki-values for PDE5-inhibition by pentoxifylline was significantly increased compared to normothermia (IC_50_: 39.4 µM ± 10.9 µM vs. 7.70 µM ± 0.265 µM, Ki: 10.0 ± 2.78 vs. 1.95 ± 0.067, *p*-value = 0.011).

### Cellular efflux

At temperatures below 28 °C, we observed no inhibition of cAMP-efflux and thus no results below 28 °C are presented.

**Fig. 3:**
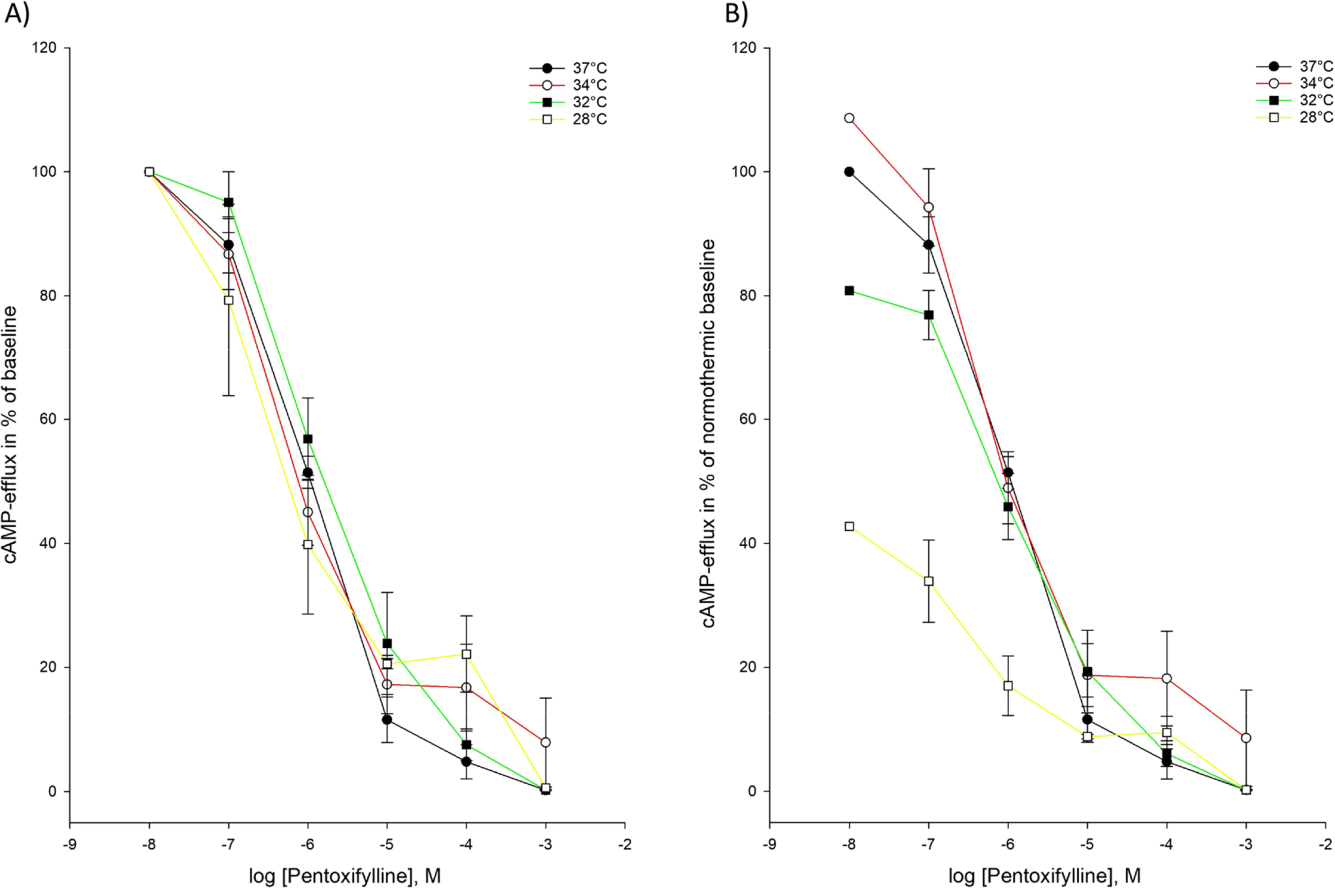
Temperature-dependent inhibition of cAMP-efflux by pentoxifylline **A** Pentoxifylline inhibition curves for cAMP-efflux activity at temperatures ranging from 37 to 20 °C. The doses of pentoxifylline are in logarithm of the concentration in mol/L. **B** Inhibition curves for cAMP-efflux activity by pentoxifylline in % of normothermic inhibition curve at temperatures ranging from 37 to 20 °C. The doses of pentoxifyllne are in logarithm of the concentration in mol/L

**Fig. 4:**
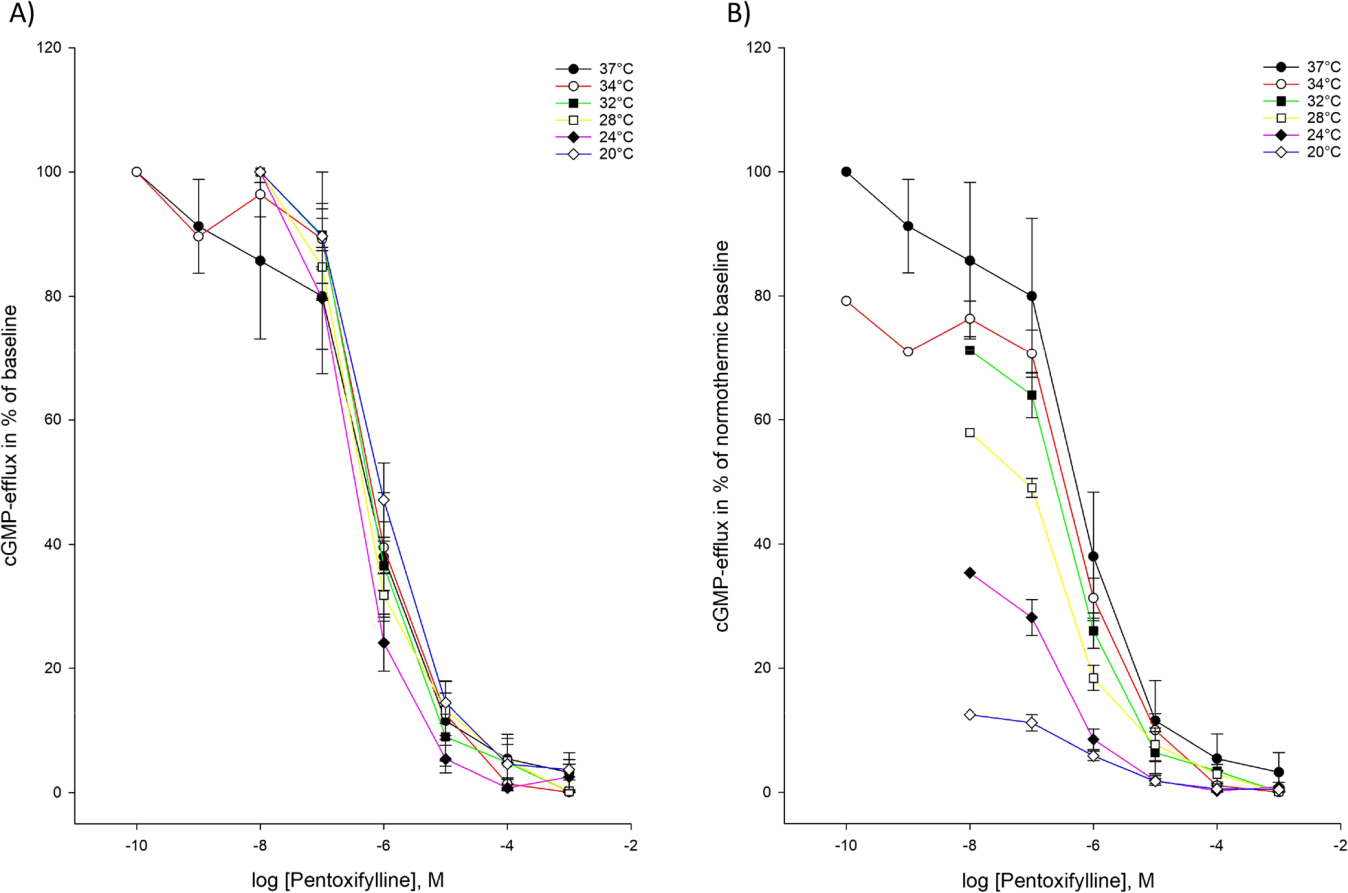
Temperature-dependent inhibition of cGMP-efflux by pentoxifylline **A** Pentoxifylline inhibition curves for cGMP-efflux activity at temperatures ranging from 37 to 20 °C. The doses of pentoxifylline are in logarithm of the concentration in mol/L. **B** Inhibition curves for cAMP-efflux activity by pentoxifylline in % of normothermic inhibition curve at temperatures ranging from 37 to 20 °C. The doses of pentoxifyllne are in logarithm of the concentration in mol/L

Pentoxifylline was able to inhibit both cAMP-efflux and cGMP-efflux at all temperatures included in our hypothermia protocol. The observed IC_50_-values and Ki-values at different temperatures showed no statistically significant changes during hypothermia compared to normothermia for either cAMP- or cGMP-efflux (Tables 2, 3) (Figs. 3, 4).

### Drug selectivity

Comparison between IC_50_-values at different temperatures were performed to evaluate drug selectivity changes during hypothermia. (Table 4). The ratio between PDE3-inhibition/PDE5-inhibition, showed an apparent decrease during reduction in temperature from 2.05 at 37 °C to 0.788 at 20 °C. The ratio between IC_50_-values for cAMP-efflux inhibition/cGMP-efflux inhibition, remained stable from 2.50 at 37 °C to 2.27 at 28 °C. The ratio between PDE3-inhibition/cAMP-efflux inhibition also remained stable during cooling (11.9 at 37 °C and 11.6 at 28 °C). The ratio between IC_50_ for PDE5-inhibiton/cGMP-efflux inhibition increased during hypothermia from 14.5 at normothermia to 102 at 20 °C, meaning that inhibition of cGMP elimination by pentoxyfilline is increasingly dependent on efflux-inhibition at low temperatures.

**Table 4:** Values depicting selectivity of pentoxifylline for the different elimination pathways of cAMP and cGMP at temperatures ranging from 37 to 20 °C

Temperature	[PDE3-inhibition]/[PDE5-inhibition]	[cAMP-efflux inhibition]/[cGMP-efflux inhibition]	[PDE3-inhibition]/[cAMP-efflux inhibition]	[PDE5-inhibition]/[cGMP-efflux inhibition]
37 °C	2.05	2.50	11.9	14.5
34 °C	1.33	4.04	3.94	12.0
32 °C	1.72	2.78	13.3	21.4
28 °C	0.920	2.27	11.6	28.6
24 °C	1.16	–	–	50.2
20 °C	0.788	–	–	102

Values are ratios between IC_50_-values

## Discussion

The present study shows that pentoxifylline has sustained effects on inhibiting elimination of cAMP and cGMP, through both inhibiting enzymatic breakdown and cellular efflux, at temperatures down to 20 °C. The only significant difference from baseline (37 °C) was detected at 20 °C for PDE5-inhibition.

A large review on cardiovascular effects of pentoxifylline from 2016 suggests the clinical serum concentrations to be in the 1–10 µM range [24]. This corresponds to most of the IC_50_-values from our study, indicating that pentoxifylline may effectively inhibit both cAMP-elimination and cGMP-elimination through PDE- and efflux inhibition during normo- and hypothermia. Inhibiting both cAMP and cGMP elimination could prove promising as both increased inotropy and increased vasodilation have alleviated HCD in vivo [8, 25]. Patients suffering from hypothermia show a high mortality rate and patient studies suggest that decreased cardiac function [19, 26] and altered hemodynamics play a role in the pathophysiology. While difficult to measure in humans suffering from hypothermia, the beforementioned in vivo experiments suggest decreased CO and increased TPR among the underlying mechanisms, causing HCD.

Increased TPR can also be a complication to veno-arterial extra-corporeal membrane oxygenation (VA-ECMO) [27], the gold-standard treatment of accidental hypothermia victims that require cardiovascular support during rewarming [1, 19]. During the VA-ECMO treatment when venous blood is drawn from the patient, pumped through an oxygenator and back to the arterial side, some blood still runs through the atriums, pulmonary circulation, and ventricles of the heart. As the VA-ECMO increases the blood flow and blood pressure, a common complication is left ventricle (LV) failure, as the LV has to overcome increased afterload in order to eject blood through the aortic valve [27]. This, in turn, leads to decreased cardiac output and makes it problematic to wean patients off the VA-ECMO when rewarmed. Strategies to overcome this VA-ECMO induced LV-failure include inotropes and vasodilators [27]. As IC_50_-values for PDE3- and PDE5-inhibition by pentoxifylline show similar concentrations both during hypothermia and normothermia, it is possible that administration of pentoxifylline in hypothermic patients on VA-ECMO can be beneficial as it provides both increased inotropy and vasodilation.

Traditionally, pentoxifylline is known as a drug used to treat patients with intermittent claudication [28]. Among the underlying mechanisms for its benefits is increased cAMP in erythrocytes, leading to increased flexibility of the erythrocytes and decreased blood viscosity [24]. The reduced viscosity eases blood passage past the atherosclerotic plaques in intermittent claudication patients, increases peripheral blood flow and thus reduces leg pain. The same decrease in blood viscosity caused by pentoxifylline might also be useful for hypothermic patients. During hypothermia, blood viscosity increases and decreased organ specific blood flow is observed [29]. By lowering the blood viscosity, organ specific blood perfusion may increase as the erythrocytes become more flexible and increases the ability to reach organs throughout the body [30]. In vivo, a combination of inotropic support and vasodilation has shown promising effects for increasing the organ specific blood flow during hypothermia and rewarming [25]. This effect was caused by a PDE3-inhibitor, levosimendan, which increases cAMP in both cardiac muscle and vascular smooth muscle. As these effects could be achievable with administration of pentoxifylline, the additional benefit of reduced blood viscosity advocates further assessment for use in hypothermic patients.

Pentoxyfilline could however cause adverse effects, such as arrythmias and altered blood pressure [31]. Such complications are not uncommon in hypothermia per se [29], and it is possible that administering pentoxifylline to hypothermic patients, could lead to increased risk. Both in vivo, ex vivo and human data show that there is an increased risk of ventricular fibrillation (VF) in hypothermia, when the core temperature is around 30 °C [32–34]. Potential aggravation of such lethal side effects should be further assessed in the experimental setting before clinical implementation of pentoxifylline for treatment of hypothermic patients is considered.

## Conclusion

IC_50_-values for cAMP- and cGMP-elimination appear similar for pentoxifylline in hypothermic conditions. This is clinically relevant as both increased inotropy and vasodilation through intracellular cAMP and cGMP increase are promising pathways to treat hypothermic patients and prevent HCD. It is, however, important to assess the physiological and pharmacokinetic properties, as well as risk of possible side effects, to evaluate the safety of pentoxyfilline in hypothermic patients.

## Data Availability

The datasets used and/or analysed during the current study are available from the corresponding author on reasonable request.

## Abbreviations

HCD: Hypothermia-induced cardiac dysfunction
TTM: Targeted temperature management
CO: Cardiac output
TPR: Total peripheral resistance
PDE: Phosphodiesterase
cAMP: Cyclic adenosine monophosphate
cGMP: Cyclice guanosine monophosphate
ABCC: ATP-binding cassette, subfamiliy C
MS: Mass spectrometry
IOV: Inside-out vesicle
VA-ECMO: Veno-arterial extra-corporeal membrane oxygenation
LV: Left ventricle
