# Correction: Pharmacodynamic properties for inhibition of cAMP- and cGMP elimination by pentoxifylline remain unaltered in vitro during hypothermia

**DOI:** 10.1186/s13049-024-01252-8

**Published:** 2024-09-04

**Authors:** Anders Lund Selli, Adrina Kalasho Kuzmiszyn, Natalia Smaglyukova, Timofey Kondratiev, Ole-Martin Fuskevåg, Georg Sager, Erik Sveberg Dietrichs

**Affiliations:** 1 Department of Medical Biology, Experimental and Clinical Pharmacology, UiT – The Arctic University of Norway, Tromsø, Norway; 2 Research and Development Department, Norwegian Air Ambulance Foundation, Oslo, Norway; 3 Center for Psychopharmacology, Diakonhjemmet Hospital, Oslo, Norway; 4 Anesthesia and Critical Care Research Group, Department of Clinical Medicine, UiT - The Arctic University of Norway, Tromsø, Norway; 5 Division of Diagnostic Services, Department of Laboratory Medicine, University Hospital of North Norway, Tromsø, Norway; 6 Division of Surgical Medicine and Intensive Care, University Hospital of North Norway, Tromsø, Norway

**Correction to: Selli et al. Scand J Trauma Resusc Emerg Med (2022) 30:73**

10.1186/s13049-022-01060-y

Following the publication of the Original Article, the author reported an error in the Methods section. The authors wrote “…containing [13 C,2H3]-Pentoxifylline, 250 nM (Alsachim, Illkirch Graffenstaden, France).”

The correct concentration was 50 nM, so it should read “…containing [13 C,2H3]-Pentoxifylline, 50 nM (Alsachim, Illkirch Graffenstaden, France).”

The error has no consequence on the results or interpretations.

The Original Article has been corrected.

